# An epidemiological study of unintentional pediatric firearm fatalities in the USA, 2009–2018

**DOI:** 10.1186/s40621-023-00438-5

**Published:** 2023-06-26

**Authors:** Arti Vaishnav, Gary A. Smith, Jaahnavi Badeti, Nichole L. Michaels

**Affiliations:** 1 The Ohio State University College of Medicine, Columbus, OH USA; 2 Center for Injury Research and Policy, The Abigail Wexner Research Institute at Nationwide Children’s Hospital, 700 Children’s Drive, Columbus, OH 43205 USA

**Keywords:** Firearm, Pediatric, Mortality, Unintentional, Injury, Violence, Surveillance

## Abstract

**Background:**

Firearm injuries are the leading cause of mortality among children and adolescents 1–19 years old in the USA. Many prior studies on this topic lack detailed information about the circumstances of the firearm fatalities and include decedents and shooters of all ages. This study characterizes firearm fatalities in the USA in which children < 15 years old unintentionally killed themselves or another child.

**Methods:**

Ten years of data from the National Violent Death Reporting System were analyzed. Unintentional firearm fatalities among children were reviewed to identify characteristics of decedents and the children who inflicted the deaths, their relationship, and circumstances of the deaths. There were 279 firearm fatalities during the study period involving children < 15 years old who unintentionally killed themselves or another child < 15 years old.

**Results:**

Most victims were male (81.4%), and 40.9% were 2–4 years old. Most incidents (64.0%) occurred at the victim’s residence, and in 80.9% of cases the firearm owner was a relative of the shooter. In the < 5-year age group, 80.3% of injuries were self-inflicted, and in the 10–14-year age group, 32.3% of shooters were a friend of the victim.

**Conclusion:**

This study highlights that children in the USA are shooting themselves and each other in their own homes, and often accessing firearms owned by family members. These findings can be used to guide prevention efforts, such as child access prevention laws, to reduce the number of pediatric firearm fatalities in the future.

## Background

Firearm violence is an important public health problem in the USA that affects the pediatric population. Firearm-related injuries are now the leading cause of death among children and adolescents 1–19 years of age (Goldstick et al. 2022; Centers for Disease Control and Prevention. Web-based Injury Statistics Query and Reporting System (WISQARS) Fatal Injury Reports. National Center for Injury Prevention and Control, Centers for Disease Control and Prevention 2022). Across 29 high-income countries, 96.7% of children < 5 years old and 92% of children and adolescents 5–14 years old who died by firearms died in the USA (Grinshteyn and Hemenway 2019). And for every child who dies from a firearm injury in the USA, is it estimated that 2.5 times as many are treated in an emergency department for a non-fatal firearm injury (Kaufman et al. 2021). It is estimated that initial hospitalizations for pediatric firearm injuries cost the US $109 million annually, not including additional expenses for hospital readmissions and other related costs (Taylor et al. 2021; Quiroz et al. 2020). The impact of firearm injuries can be long-lasting, both physically and mentally. One study found that nearly 50% of children admitted to the hospital for a firearm-related injury were discharged with a disability (DiScala and Sege 2004). A second study found that more than one-quarter of children and teens with a non-fatal firearm injury were diagnosed with a new mental health disorder within 12 months of their injury (Oddo et al. 2021). These figures do not account for the impact of secondary exposure to firearm violence on the wellbeing of children who are not physically injured by guns (Ranney 2019).

A study by Wintemute et al. published in 1987 was one of the first to epidemiologically describe pediatric firearm injuries in a US state (Wintemute et al. 1987). Wintemute and colleagues used California death certificate data to examine firearm fatalities in which both the decedent and shooter were younger than 15 years old (Wintemute et al. 1987). For the current research, we build on the study design of Wintemute et al. by using multi-state data from the NVDRS to describe cases in which children < 15 years old were unintentionally shot and killed by other children or themselves.

Prior studies have primarily focused on firearm fatalities based on the age of the victim. Some studies focused on victims of any age (Hemenway et al. 2010; Solnick and Hemenway 2019), while others limited the study population to victims who are children (Bleyer et al. 2021). However, in all these studies, shooters are of any age. We chose to look at the unique circumstances of unintentional firearm fatalities that happen within the pediatric population. Unintentional firearm injuries are much less common than firearm-related homicides and suicides, and unlike intentional firearm injuries, these incidents often involve young children. Therefore, the strategies needed to prevent these injuries may differ from those designed to prevent injuries among older children. Also, because these injuries often result from one child shooting another child, the wellbeing of the child who shot the gun but was physically unharmed must also be considered when thinking about treatment and follow up care after these incidents.

Previous research on unintentional firearm deaths has used data from a variety of sources, including trauma centers, police reports, county medical examiner records, and the National Vital Statistics System (Wintemute et al. 1987; Grossman et al. 1999, 2005; Leventhal et al. 2014; Fowler et al. 2017). These datasets often provide limited information on the victim, the shooter(s), or the details of the injury events, and under- and overreporting of unintentional firearm fatalities is common (Barber and Hemenway 2011). Some recent studies of firearm deaths have used the National Violent Death Reporting System (NVDRS), an incident-based injury surveillance system maintained by the Centers for Disease Control and Prevention (CDC). Compared to other data sources, the NVDRS more accurately captures unintentional firearm fatalities and provides a deeper understanding of violent deaths through a comprehensive set of variables and case narratives and describes the characteristics of both shooter(s) and victim(s) (Hemenway et al. 2010; Solnick and Hemenway 2019; Barber and Hemenway 2011; Hemenway and Solnick 2015).

By analyzing ten years of NVDRS data, we expand upon prior research by using a more recent, larger, and geographically diverse sample. We describe the epidemiological characteristics of fatal firearm incidents in which children unintentionally killed themselves or other children and compare the circumstances of these deaths between different demographic groups. We also examine the findings by age group to consider the role that child development may have in predicting and preventing these injuries.

## Methods

### Data source

This cross-sectional study was conducted using the NVDRS Restricted Access Dataset for the years 2009 through 2018. The NVDRS is a state-based surveillance system that combines data from multiple sources, including coroner/medical examiner and law enforcement reports and death certificates to provide a comprehensive understanding of fatalities (Centers for Disease Control and Prevention. NVDRS frequently asked questions. National Center for Injury Prevention and Control, Division of Violence Prevention. 2022). A variety of information is collected about both victims and suspects, including demographics, mental health status, toxicology reports, weapons used, and other relevant data. The NVDRS also provides narrative summaries of cases based on coroner/medical examiner and law enforcement reports.

The NVDRS began collecting data from 6 states in 2003, and additional states have joined the program at various times since then. Forty-one states contributed data to the NVDRS during the years we studied; however, only 33 states reported cases that met the criteria for this study. Due to differences in when states began contributing data to the NVDRS, some states provided less than 10 years of data to the current study. States contributing data that met our study criteria included: Alaska, Colorado, Georgia, Kentucky, Maryland, Massachusetts, New Jersey, New Mexico, North Carolina, Oklahoma, Oregon, South Carolina, Utah, Virginia, and Wisconsin, (2009–2018); Ohio (2011–2018); Michigan (2014–2018); Arizona, Kansas, Maine, Minnesota, and New York (2015–2018); Illinois, Indiana, Iowa, Pennsylvania, and Washington (2016–2018); Delaware and Nevada (2017–2018); Alabama, Louisiana, Missouri, and Nebraska (2018). Illinois, Pennsylvania, and Washington collected data on > 80% of violent deaths in their states, per requirements specific to their funding.

### Case selection criteria

**Fig. 1:**
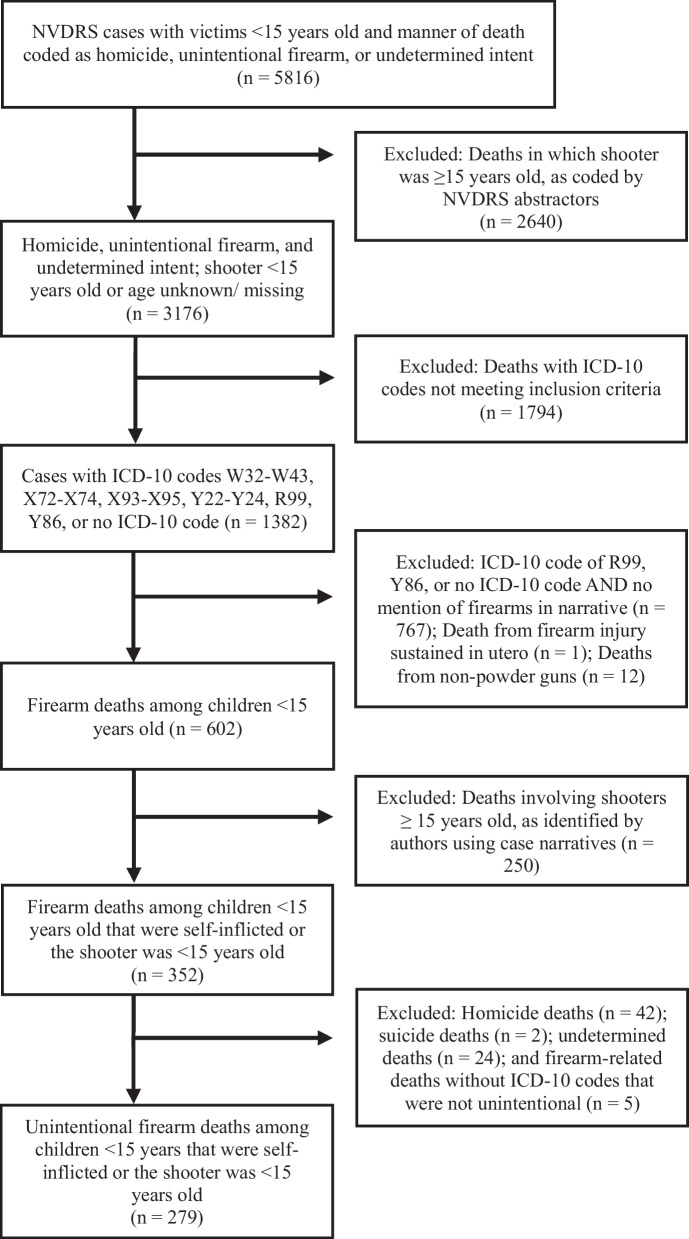
Flow chart depicting case selection process

Data were obtained from the CDC for all fatalities in the NVDRS that (1) involved children < 15 years old and (2) had an abstractor coded manner of death that was recorded as homicide, unintentional firearm death, or undetermined intent. In order to identify all cases in which a child < 15 years old was unintentionally shot and killed by another child < 15 years old or themselves, we conducted a multi-step case selection process (Fig. 1). From prior work with the NVDRS and previous research on firearm injuries among children, we anticipated that some cases meeting our inclusion criteria could be miscoded (e.g., categorized as homicides instead of unintentional deaths), so we chose to include an extensive review of case narratives as part of our case selection process.

First, the dataset was filtered to remove all cases in which the suspected shooter was ≥ 15 years old. (Cases in which the person who shot the gun’s age was missing or unknown were kept in the dataset until later in the process.) We then identified firearm-related fatalities using the following International Classification of Diseases, 10th Revision (ICD-10) codes for underlying cause of death: W32-W43 (unintentional firearm deaths), X72-X74 (firearm suicides), X93-X95 (firearm homicides), and Y22-Y24 (firearm deaths of undetermined intent). For cases in which no ICD-10 code was provided or the ICD-10 code was R99 (ill-defined and unknown cause of mortality), or Y86 (sequelae of other accidents), a member of the study team reviewed the coroner/medical examiner and law enforcement narratives to identify firearm-related fatalities. If the case narrative did not indicate that the death was related to firearms, the case was removed from the dataset. While reviewing these narratives for firearm-related fatalities, we also removed 1 death from a firearm injury sustained in utero and 12 fatalities related to non-powder guns (e.g., BB guns).

Next, a member of the study team reviewed the narratives of all cases in which the variable corresponding to the age of the person who shot the gun was unknown or missing. Cases were removed from the dataset if the person who shot the gun was determined to be ≥ 15 years old or if the identity or age of the shooter could not be determined due to lack of information or ambiguity in the case narratives. In cases of discrepancies between the CME and LE narratives regarding the age of the shooter, if both narratives indicated that the shooter was < 15 years old, the case was included and the child’s age was coded as “< 15 years”. When the age of the shooter was not coded by NVDRS abstractors or included in the narratives, context clues were used to identify cases meeting the inclusion criteria. For example, if a 10 year-old child was shot by a “younger sibling”, but the age was not given, the case was included in the study and the child who shot the gun was coded as age “< 15 years”. Similarly, if two toddlers, ages 2 and 3 years, were left alone with a gun and the narrative(s) indicated that it could not be determined whether the decedent shot himself or was shot by the other child, the case was included in the study, even though the exact age of the child who shot the gun could not be determined.

Lastly, two members of the study team reviewed the remaining narratives for deaths in which the ICD-10 code indicated the underlying cause of death was firearm suicide (*n* = 6), firearm homicide (*n* = 99), or firearm death of undetermined intent (*n* = 36) and reclassified any cases in which the incident was clearly unintentional. (Although we did not request cases in which the manner of death was suicide, the dataset we received did contain 6 cases with an ICD-10 code indicating suicide, which we reviewed further to determine whether they met our study criteria.) The keywords and characteristics used to indicate the death was unintentional can be found in Table 1. Cases were deemed to be unintentional when narratives included keywords and phrases such as “accidental”, “thought gun was a toy”, “thought gun was unloaded”, or “playing with the gun”. One frequent scenario in which cases were coded as a homicide in the NVDRS system but recoded as an unintentional death by the study team were cases in which a child shot another child, but an adult in the home was charged with homicide for not properly storing the firearm away from children. Narratives of cases that were originally coded as unintentional in the NVDRS were also reviewed to ensure accuracy. After recoding was complete, the final dataset included only unintentional firearm fatalities in which one child < 15 years old killed another child < 15 years old, or in which a child < 15 years old shot themselves.

**Table 1:** Keywords or circumstances in narratives of fatalities categorized as unintentional

“Unintentional”
“Accident”
“Playing”
Child who fired gun took magazine out
Child thought gun was unloaded
Child thought firearm was a BB gun
Child thought gun was a toy
Child was handling firearm recklessly when it discharged
Child who fired gun had developmental delay and no intent to harm victim
Child who fired gun was < 6 years old
Child was charged with involuntary/negligent manslaughter
Gun slipped out of the child’s hands

Throughout the coding process, cases were omitted if there was little to no relevant information in the case narratives or if the circumstances of the case could not be determined after reading the case narratives and NVDRS abstractor classifications. All cases included in the final dataset had either a coroner/medical examiner narrative, a law enforcement narrative, or both. For cases in which the case narratives of the coroner/medical examiner and law enforcement differed, the NVDRS abstractor classification of the death was used. Disagreements within the study team about the coding of cases were settled through review and discussion until consensus was reached.

### Measures

To better categorize cases, case narratives were used to complete missing data for the variables ‘suspect age in years’, ‘suspect sex’, ‘victim to suspect relationship’, and ‘injury location type’, when the data were present in the narratives but not coded in the dataset. The ‘victim to suspect relationship’ and ‘injury location type’ variables were provided in the NVDRS database; however, we recategorized these variables for analyses. The ‘victim to suspect relationship’ variable was recoded to categorize the child who shot the firearm as one of the following: (1) brother (including half-brother and stepbrother), (2) sister (including half-sister and stepsister), (3) sibling, sex unknown, (4) other relative (e.g., child’s uncle or cousin), (5) self, (6) friend, (7) other, and (8) unknown. The ‘injury location type’ variable was recoded as: (1) victim’s residence, (2) relative of victim’s residence, (3) natural area (e.g., field), (4) friend of victim’s residence, (5) other residence, (6) motor vehicle, (7) commercial establishment, and (8) unknown. While the NVDRS uses the term “suspect” in unintentional firearm deaths to describe the individual who fired the weapon, because this study involves unintentional shootings among children, we will not use that language except to describe how NVDRS variables were used or recoded for the current study. As required by the CDC, some results were combined or masked to hide small cell sizes (*n* < 5) that could potentially be used to identify decedents.

### Statistical analyses

Analyses for this study focused on examining fatality distributions by age and comparing victim/shooter characteristics and circumstances of the injury incidents by age of decedent in both self-inflicted cases and cases in which another child shot the firearm. Distributions are described using frequencies and percentages and means and standard deviations. All analyses were conducted using IBM SPSS Statistics, version 26.0 (IBM Corp., Armonk, NY). This study was determined not to be human subjects research by the Institutional Review Board at the authors’ institution.

## Results

NVDRS data from 2009 to 2018 included 170 cases coded as unintentional firearm fatalities in which both the decedent and the child who shot the firearm were < 15 years old, or in which a child < 15 years old died of an unintentionally self-inflicted firearm injury. The study team reviewed case narratives for all remaining firearm-related fatalities among the age group of interest and determined that 24 of the ‘unknown’ cases (missing or unrelated ICD-10 code), one case with an ICD-10 code of R99 (ill-defined and unknown cause of mortality), one case with an ICD code of Y86 (sequelae of other accidents), four cases labeled as suicide, 66 cases labeled as homicide, and 13 firearm deaths of underdetermined intent were clearly unintentional based on the case narratives. Thus, a total of 279 unintentional firearm fatalities from 33 states were included in the study.

### Decedent and shooter demographics

**Fig. 2:**
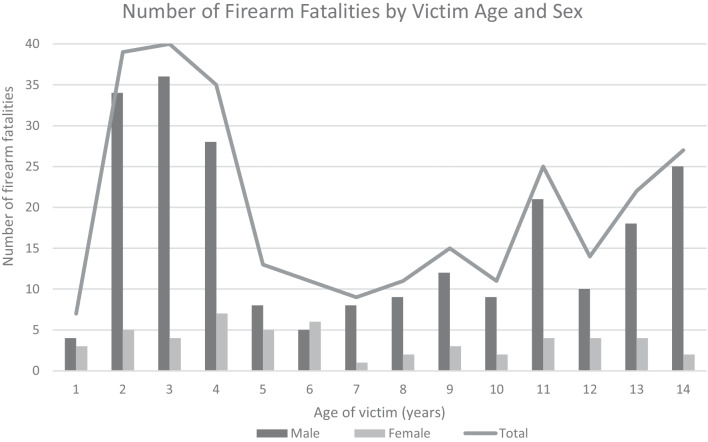
Frequency of unintentional firearm fatalities by victim age and sex, NVDRS, 2009–2018

Figure 2 shows a bimodal distribution of unintentional firearm fatalities, with 40.9% of victims between 2 and 4 years old (*n* = 114), and 31.5% of victims between 11 and 14 years old (*n* = 88). Males comprised 81.4% of victims (Table 2). Approximately one-half (50.5%) of victims were White and 39.8% were Black or African American. The majority of victims (92.7%) were non-Hispanic. Among all fatalities, 155 (56.6%) deaths were self-inflicted, while in 118 (43.1%) deaths, the shooter was another child. For 6 cases, it could not be determined whether the shooter was the decedent or another child < 15 years old who was described in the case narrative.

**Table 2:** Descriptive characteristics of victim and victim death, NVDRS, 2009–2018

Characteristics	Victim age group			
	< 5 years *n* (%)^a^	5–9 years *n* (%)^a^	10–14 years *n* (%)^a^	Total *n* (%)^a^
Victim sex				
Male	102 (84.3)	42 (71.2)	83 (83.8)	227 (81.4)
Female	19 (15.7)	17 (28.8)	16 (16.2)	52 (18.6)
Victim race				
White	58 (47.9)	24 (40.7)	59 (59.6)	141 (50.5)
Black or African American	53 (43.8)	26 (44.1)	32 (32.3)	111 (39.8)
Other/unspecified^b^	10 (8.3)	9 (15.3)	8 (8.1)	27 (9.7)
Victim ethnicity				
Not Hispanic	113 (94.2)	*	*	255 (92.7)
Hispanic	7 (5.8)	*	*	20 (7.3)
Unknown^c^	1	1	2	4
Location of shooting				
Victim’s residence	89 (74.2)	39 (66.1)	50 (50.1)	178 (64.0)
Relative's residence	14 (11.7)	7 (11.9)	7 (7.1)	28 (10.1)
Natural area (field, river, beach, woods)	0 (0.0)	5 (8.5)	14 (14.1)	19 (6.8)
Friend's residence	*	*	18 (18.2)	19 (6.8)
Other residence	7 (5.8)	5 (8.5)	5 (5.1)	17 (6.1)
Motor vehicle/commercial establishment	*	*	5 (5.1)	17 (6.1)
Unknown^c^	1	0	0	1
Location of victim death				
Emergency department/outpatient	56 (46.7)	26 (44.1)	26 (26.3)	108 (38.8)
Home	31 (25.8)	17 (28.8)	23 (23.2)	71 (25.5)
Hospital inpatient	21 (17.5)	9 (15.3)	23 (23.2)	53 (19.0)
Other^d^	12 (10.0)	7 (11.9)	27 (27.3)	46 (16.5)
Unknown^c^	1	0	0	1
Shooter relationship to victim				
Self	94 (80.3)	25 (43.1)	36 (36.4)	155 (56.6)
Brother^e^	16 (13.7)	22 (37.9)	22 (22.2)	60 (21.9)
Friend	0 (0.0)	*	32 (32.3)	*
Other relative^f^	7 (6.0)	6 (10.3)	9 (9.1)	22 (8.0)
Other^g^	0 (0.0)	*	0 (0.0)	*
Unknown^c^	4	1	0	5
Owner of firearm				
Parent of shooter	50 (71.4)	14 (50.0)	26 (53.1)	90 (61.2)
Other family member of shooter	13 (18.6)	7 (25.0)	9 (18.4)	29 (19.7)
Other^h^	7 (10.0)	7 (25.0)	14 (28.6)	28 (19.0)
Unknown^c^	51	31	50	132
Total (row %)	121 (43.4)	59 (21.1)	99 (35.5)	279 (100.0)

*Indicates cell frequency masked to suppress small cells sizes

^a^Column percentage may not total 100.0% due to rounding or masked cell values

^b^Other/Unspecified includes Asian/Pacific Islander, American Indian/Alaska Native, and two or more races

^c^Unknown values were omitted from the denominator when calculating percentages

^d^Other includes dead on arrival at health care facility and other locations

^e^Brother includes stepbrother, half-brother

^f^Other relative includes uncle, cousin, sibling sex unknown, and sister (including stepsister, half-sister)

^g^Other includes schoolmate and neighbor

^h^Other includes friend/acquaintance of shooter and shooter’s own firearm

Among the cases in which the shooter was another child, the mean age of the child who fired the weapon was 9.91 years (SD: 4.47 years), and 64.4% of these children were 10–14 years old (Table 3). Most shooters were male (92.9%), 49.5% were White, and 27.8% were Black or African American. When ethnicity data were available, 92.3% of shooters were non-Hispanic (Table 3). Among self-inflicted unintentional firearm deaths, the mean age of decedents was 5.85 years (SD: 4.40 years) and 87.1% of victims were male (Table 4).

**Table 3:** Descriptive characteristics of shooters, NVDRS, 2009–2018

	Shooter age group			
	< 5 years *n* (%)^a^	5–9 years *n* (%)^a^	10–14 years *n* (%)^a^	Total *n* (%)^a^
Shooter sex				
Male	*	21 (80.8)	*	105 (92.9)
Female	*	5 (19.2)	*	8 (7.1)
Unknown^b^	1	3	1	5
Shooter race				
White	5 (50.0)	8 (36.4)	35 (53.8)	48 (49.5)
Black or African American	5 (50.0)	7 (31.8)	15 (23.1)	27 (27.8)
Other/unspecified^c^	0 (0.0)	7 (24.1)	15 (23.1)	22 (17.7)
Unknown^b^	3	7	11	21
Shooter ethnicity				
Not Hispanic	7 (100.0)	*	*	60 (92.3)
Hispanic	0 (0.0)	*	*	5 (7.7)
Unknown^b^	6	13	34	53
Total (row %)	13 (11.0)	29 (24.6)	76 (64.4)	118 (100.0)

*Indicates cell frequency masked to suppress small cells sizes

^a^Column percentage may not total 100.0% due to rounding or masked cell values

^b^Unknown values were omitted from the denominator when calculating percentages

^c^Other/Unspecified includes Asian/Pacific Islander and American Indian/Alaska Native

**Table 4:** Descriptive characteristics of victim in self-inflicted cases, NVDRS, 2009–2018

	Victim age group			
	< 5 years *n* (%)^a^	5–9 years *n* (%)^a^	10–14 years *n* (%)^a^	Total *n* (%)^a^
Sex				
Male	85 (90.4)	*	*	135 (87.1)
Female	9 (9.6)	*	*	20 (12.9)
Victim race				
White	44 (46.8)	9 (36.0)	18 (50.0)	71 (45.8)
Black or African American	40 (42.6)	15 (60.0)	14 (38.9)	69 (44.5)
Two or more races	6 (6.4)	*	*	8 (5.2)
Other/unspecified^b^	*	1 (4.0)	*	7 (4.5)
Victim ethnicity				
Not Hispanic	*	24 (100.0)	*	148 (96.7)
Hispanic	*	0 (0.0)	*	5 (3.3)
Unknown^c^	0	1	1	2
Total (row %)^a^	94 (60.6)	25 (16.1)	36 (23.2)	155 (100.0)

*Indicates cell frequency masked to suppress small cells sizes

^a^Column and row percentages may not total 100.0% due to rounding or masked cell values

^b^Other/Unspecified includes American Indian or Alaska Native and Asian/Pacific Islander

^c^Unknown values were omitted from the denominator when calculating percentages

### Characteristics of the shooting by age group

Among victims < 5 years old, approximately three-fourths (74.2%) of fatalities occurred at the victim’s residence (Table 2). In the 10–14-year age group, one-half (50.1%) of fatalities occurred at the victim’s residence, 18.2% occurred at a friend’s residence, and 14.1% occurred in a natural area.

In the < 5-year age group, 80.3% of fatalities were self-inflicted compared with 43.1% and 36.4% in the 5–9-year and 10–14-year age groups, respectively. Among cases in which the shooter was another child, the most common relationship was a brother to the victim (50.4%). Approximately one-third (32.3%) of shooters whose victims were in the 10–14-year age group were a friend of the victim, while victims in the < 5-year age group who were shot by others were most commonly shot by a brother (13.7%).

### Firearm access, circumstances, and injuries

When information was available on how the firearm used in the death was stored, most were stored loaded (*n* = 125, 91.9%) and unlocked (*n* = 129, 91.5%). For these two variables, 48.7% and 49.5% of this information was unknown or missing from the NVDRS data, respectively. Among cases in which the owner of the firearm was known (*n* = 147, 52.7%), the owner was most frequently the parent of the shooter (61.2%) or another family member of the shooter (19.7%) (Table 2). Playing with the firearm or mistaking the firearm for a toy were the most common circumstances involved in these deaths (52.7%). Most fatalities involved a handgun (72.1%), with semi-automatic pistols being the most commonly used firearm overall (37.9%, Table 5). Rifles and shotguns were involved in 43.4% of fatalities among children 10–14 years of age. Most firearms wounds were to the head/neck region (51.4%), followed by face (24.0%), and thorax/abdomen (21.1%) (Table 5).

**Table 5:** Wound location, circumstance of incident, type of firearm, NVDRS, 2009–2018

	Victim age group			
	< 5 years *n* (%)^a^	5–9 years *n* (%)^a^	10–14 years *n* (%)^a^	Total *n* (%)^a^
Location of wound^b^				
Head/neck	81 (49.4)	38 (54.3)	61 (52.6)	180 (51.4)
Face	50 (30.5)	15 (21.4)	19 (16.4)	84 (24.0)
Thorax/abdomen	28 (17.1)	16 (22.9)	30 (25.9)	74 (21.1)
Other^c^	5 (3.0)	1 (1.4)	6 (5.2)	12 (3.4)
Circumstance^d^				
Playing with firearm/firearm mistaken for toy	100 (70.9)	44 (52.4)	51 (35.2)	195 (52.7)
Unintentionally pulled trigger	22 (15.6)	7 (8.3)	20 (13.8)	49 (13.2)
Thought firearm was unloaded	2 (1.4)	10 (11.9)	21 (14.5)	33 (8.9)
Hunting/aiming for target	0 (0.0)	6 (7.1)	12 (8.3)	18 (4.9)
Showing firearm to another person	0 (0.0)	1 (1.2)	12 (8.3)	13 (3.5)
Firearm was dropped	2 (1.4)	4 (4.8)	3 (2.1)	9 (2.4)
Loading/unloading ammunition	0 (0.0)	0 (0.0)	7 (4.8)	7 (1.9)
Other^e^	15 (10.6)	12 (14.3)	19 (13.1)	46 (12.4)
Type of firearm				
Handgun, pistol-semi-automatic	56 (54.4)	19 (35.2)	16 (19.3)	91 (37.9)
Handgun, unknown type	33 (32.0)	5 (9.3)	13 (15.7)	51 (21.3)
Rifle, any type	*	*	26 (31.3)	40 (16.7)
Handgun, revolver	7 (6.8)	7 (13.0)	17 (20.5)	31 (12.9)
Shotgun, any type	*	*	10 (12.0)	24 (10.0)
Other^f^	1 (1.0)	1 (1.9)	1 (1.2)	3 (1.3)
Unknown^g^	18	5	16	39

*Indicates cell frequency masked to suppress small cells sizes

^a^Column percentage may not total 100.0% due to rounding or masked cell values

^b^Multiple wounds could be present for each victim

^c^Other includes upper extremity, lower extremity, and spine

^d^Multiple circumstances could have occurred for each victim

^e^Other includes cleaning, repairing, assembling, or disassembling firearm; thought safety was on; and placed in or removed from holster or clothing

^f^Other includes: handgun, pistol–derringer; long gun, unknown type; and other

^g^Unknown values were omitted from the denominator when calculating percentages

## Discussion

In this study, we identified 279 cases of unintentional firearm fatalities in the USA occurring between 2009 and 2018 in which the injury was self-inflicted by a child < 15 years old or in which both the decedent and child who shot the gun were < 15 years old. Although the NVDRS has been shown to provide more accurate and detailed data on this topic than the National Vital Statistics System (Barber and Hemenway 2011), we identified more unintentional firearm deaths among our population of interest than were coded by NVDRS, increasing the size of our dataset by 64%.

Males accounted for more than 80% of victims and more than 90% of shooters when the shooting involved another child. Prior studies of unintentional firearm injuries and deaths among children also show that males are disproportionately represented as both victims and shooters (Fowler et al. 2017; Hemenway and Solnick 2015). Our findings highlight that these differences are significant even among very young children < 5 years old. More research is needed to understand the cultural and social influences, as well as parenting attitudes and practices, that cause such a disparity between boys and girls as young as 2 years old. While boys may be more frequently exposed to toy guns and pretend play involving guns, it is also worth exploring whether there are differences in parenting attitudes and practices that impact boys’ ability to access real firearms and how prevention efforts might be better tailored to this population.

More than half of the fatalities in this study were self-inflicted and among the self-inflicted cases, the majority occurred in the < 5-year age group. These findings are similar to previous studies that showed the majority of unintentional firearm deaths are self-inflicted in the 2–4-year age group (Wintemute et al. 1987; Hemenway and Solnick 2015). Likewise, similar to prior research, our study found that among children 5–14 years old, the majority of deaths were inflicted by another individual (Wintemute et al. 1987; Hemenway and Solnick 2015). As mentioned in Background, an important component of our study is that it also highlights a population that is often overlooked, namely children who unintentionally shoot other children. Support for these children and their families should be considered when developing response and follow up systems of care for pediatric firearm victims.

Most shootings occurred at the victim’s residence. Victims who were 10–14 years of age were also killed at friends’ homes, but this was not identified as a shooting location among younger victims. In cases in which the owner of the firearm was identified, the owner was most frequently a parent or other family member of the shooter, and many of the firearms were stored loaded. These results indicate that children are accessing firearms at a home, often their own home, and many of these firearms are not stored safely, even when there are young children in the home. These study findings also highlight that children are accessing firearms belonging to other relatives, not just their parents.

The findings of this study are consistent with prior research demonstrating that the most common circumstance for unintentional firearm deaths among children is for a child to play with a firearm or mistake a firearm for a toy (Solnick and Hemenway 2019). Parents often believe that their child knows not to pick up a firearm if they find one or mistakenly believe their child can differentiate between real firearms and toy firearms (Jackman et al. 2001; Connor and Wesolowski 2003). However, data from the current study suggest that children continue to die from injuries sustained while playing with firearms or mistaking real firearms for toys. Educational programs that instruct children how to behave when they encounter a firearm have not been shown to reliably improve firearm safety skills or prevent injuries (Crossen et al. 2015; Michaels and Smith et al. 2021). Yet, one-third of US households with children have firearms, and less than one-third of those firearm-owning households with children store their firearms unloaded and locked (Azrael et al. 2018).

These findings should be considered when creating policies and regulations to prevent firearm-related deaths among children. For example, policies that mandate gun locks and safe storage have been associated with a reduction in adolescent firearm-related suicides and unintentional firearm fatalities among young children (Connor and Wesolowski 2003; Santaella-Tenorio et al. 2016). Additionally, child access prevention laws that penalize adults for unsafe storage of firearms may be effective in limiting how often children encounter firearms in their home and other residences. In the USA, 15 states have child access prevention laws to hold firearm owners liable if a minor accesses their firearm (Everytown for Gun Safety. Secure Gun Storage. 2023). Negligence laws, which hold firearm owners liable for the unsafe storage of firearms, have been associated with significant reductions in pediatric unintentional firearm fatalities (Kivisto et al. 2021). The strictest negligence laws were associated with the greatest reduction in unintentional firearm fatalities among children < 15 years old (Kivisto et al. 2021; Azad et al. 2020). However, recklessness laws, which hold firearm owners liable for directly providing firearms to minors, have not been associated with changes in pediatric firearm fatality rates (Azad et al. 2020).

Physicians, especially pediatricians, should use screening tools and motivational interviewing to counsel families on safe firearm storage. This should be done as a routine part of their anticipatory guidance, similar to how physicians provide counseling on car seat safety and bike helmet safety. The American Academy of Pediatrics recommends counseling parents on the dangers of firearms in the home and firearm safety (Dowd and Sege 2012). Prior research has demonstrated that combining safety counseling with the distribution of firearm cable locks significantly increases safer firearm storage (Carbone et al. 2005; Barkin et al. 2008). A study by Barkin et al. found that a brief in-office intervention delivered by primary care practitioners to caregivers of children 2–11 years old at well-child visits, paired with no-cost firearm cable locks, significantly increased safe firearm storage (Barkin et al. 2008). Positive results were also found when implementing a similar program in a clinic in a predominantly Hispanic community (Carbone et al. 2005). These findings suggest the imperative role physicians can play in the prevention of pediatric firearm injuries. However, barriers such as lack of time, resources, and provider education, as well as cultural norms, may prevent physicians from implementing such interventions (Ketabchi et al. 2021; Hinnant et al. 2021).

There are limitations to this study. Data provided by NVDRS for this study reported fatalities from 33 states, and thus, data that fit our criteria are missing from the remaining states and our data may not be generalizable to the entire USA. Because of the small sample size and states joining NVDRS in different years during the duration of this study, we did not calculate trends over time. The relatively small sample size also limits reporting specific frequencies in the results due to small cell size restrictions. Future research on this topic will benefit from the recent expansion of NVDRS data collection to include all US 50 states, the District of Columbia, and Puerto Rico. This will allow researchers to more accurately estimate the number of children who are unintentionally shot and killed by other children or themselves and easily track national trends over time.

NVDRS abstractors are limited to the information that is included in investigative reports and do not have the means to correct incomplete or inaccurate reports. Therefore, some cases had missing or inconsistent narratives. In addition, some NVDRS variables are manner specific and are not collected for all manners of death. When reviewing homicides, suicides, unknown cases, and firearm fatalities of undetermined intent, the lack of information as well as discrepancies in some narratives made it difficult to determine the circumstance of the death and therefore, those cases were omitted. This may have resulted in an underestimation of unintentional firearm fatalities among children in this study. Lastly, some NVDRS variables, such as those describing how the firearm was stored, were missing data for approximately 50% of the cases included in this study. These data could have provided important information in our study, such as how the firearms were accessed.

## Conclusion

Children shooting children is an important public health issue in the USA. This study demonstrates that the circumstances of death differ by child age, with self-inflicted deaths predominating among children younger than 5 years old and shootings inflicted by another child more common among older child victims. This study highlights how firearms are accessible to children in their own homes, and the homes of relatives and friends, and are often owned by a family member of the shooter. Implementing laws and regulations to limit firearm access to children and creating new strategies to prevent these premature deaths is crucial in keeping children safe and healthy.

## Data Availability

The dataset analyzed during the current study is available by request via the NVDRS Restricted Access Database application process: https://www.cdc.gov/violenceprevention/datasources/nvdrs/dataaccess.html

## Abbreviations

CDC: Centers for Disease Control and Prevention
NVDRS: National Violent Death Reporting System
SD: Standard deviation
USA: United States of America
